# Ultrasound-Guided Versus Fluoroscopy-Guided Caudal Epidural Steroid Injection for the Treatment of Unilateral Lower Lumbar Radicular Pain: Case-Controlled, Retrospective, Comparative Study

**DOI:** 10.1097/MD.0000000000002261

**Published:** 2015-12-18

**Authors:** Ki Deok Park, Tai Kon Kim, Woo Yong Lee, JaeKi Ahn, Sung Hoon Koh, Yongbum Park

**Affiliations:** From the Department of Rehabilitation Medicine, Gachon University, Gil Medical Center, Incheon, Korea (KDP); Department of Rehabilitation Medicine, Hanyang University College of Medicine, Seoul, South Korea (TKK); Department of Anesthesiology, Sanggye Paik Hospital, Inje University College of Medicine (WYL); and Department of Rehabilitation Medicine, Sanggye Paik Hospital, Inje University College of Medicine, Seoul, Korea (JA, SHK, YP).

## Abstract

The aim of the article is to investigate the efficacy of ultrasound (US)-guided Caudal Epidural Steroid Injection (CESI) compared with fluoroscopy (FL)-guided CESI in patients with unilateral lower lumbar radicular pain.

This case-controlled, retrospective, comparative study was done at the university hospital. A total of 110 patients treated with US- or FL-guided CESI were administered a mixture of 20 cc (0.5% lidocaine 18.0 mL + dexamethason 10 mg 2 mL). Outcome measurement was assessed by Oswestry Disability Index (ODI), verbal numeric pain scale (VNS) before injections and at 3, 6, and 12 months after the last injections. Successful outcome was defined as measured by >50% improvement in the VNS score and >40% improvement in the ODI.

ODI and VNS showed improvement at 3, 6, and 12 months after the last injection in both groups. No statistical differences in ODI, VNS were observed between groups (*P* < 0.05). No significant differences in the proportion of patients with successful treatment were observed between the groups from the 3-month to 6-month to 12-month outcomes.

US-guided CESI is deserving of consideration in conservative management of unilateral lower lumbar radicular pain.

## INTRODUCTION

Caudal epidural steroid injection (CESI) can be helpful in the symptomatic treatment of lumbar radicular pain due to spinal canal stenosis or herniated disc.^1,2^ Successful caudal injection relies on the accurate placement of a needle into the epidural space through the sacral hiatus.^3^ Incorrect needle placement occurs at a frequency of 25% to 36% of cases when performed without fluoroscopy (FL) guidance.^4^ Studies on FL suggested that CESI should proceed under FL guidance and contrast media.^4–6^ However, the application of FL requires careful consideration due to the possibility of ionizing radiation exposure. Because the injection is administered close to the gonadal area, treatment of patients of reproductive age should be considered with caution.^7^ And the high cost of FL must be considered.

Chen et al^3^ showed the practicability of ultrasound (US) in locating the sacral hiatus and guiding the needle into the caudal epidural space. Chen et al^3^ also described using FL after contrast injection to confirm the position of a caudal needle placed under US guidance and reported a 100% success rate. Blanchais et al,^8^ who assessed the safety of US-guided CESI, reported that no cerebrospinal fluid reflux occurred. In addition, heme with aspiration was noted in 9/29 patients and resolved in 8 upon needle repositioning. No complications were recorded during the first month.^8^ In terms of treatment effect, Park et al^9^ demonstrated that US- and FL-guided CESI provided adequate pain reduction and improvement of function for 3 months when performed in patients with unilateral lower lumbar radicular pain, and no statistical differences in the verbal numerical rating scale (VNS), the Oswestry Disability Index (ODI), or the effectiveness of the procedure were observed between groups.

In previous studies, only the location of the needle, safety, and short-term treatment effect were observed. One of the main limitations of US-guided CESI is that we cannot confirm that medication is delivered to the exact target site. And this limitation can have an influence on long-term treatment effects. Hence, the aim of this study was to evaluate long-term pain reduction and functional improvements of US-guided CESI compared with FL-guided CESI in the case of unilateral lower lumbar radicular pain through a retrospective study with 1-year follow-up.

## MATERIALS AND METHODS

### Study Design

This study is a retrospective comparative study of chart review. All the patients’ privacy and data were maintained confidentially throughout the research process. No direct contact with the study population was included in this study, and all patient identifiers were removed from the data set on initial collection. Approval from the Institutional Review Board of Sanggye Paik hospital was obtained including a waiver of informed consent.

## SUBJECT

Between January 2012 and December 2013, 362 patients with unilateral lower lumbar radicular pain due to spinal canal stenosis or herniated disc were referred to our pain clinic. Diagnosis of unilateral lower lumbar radicular pain was based on the clinical pain profiles, physical examinations, and CT or MRI. Clinical pain profiles mean lancinating and traveled along the length of the lower limb, in a band no more than 2 to 3 inches wide. The EMG test was used to rule out other disease such as other peripheral neuropathy, progressive motor deficit or significant sensory deficit, and cauda equina syndrome, and so on.

Those who met the following inclusion criteria were selected: aged 18 or older, received US- or FL-guided CESI. Further inclusion criteria included patients who had experienced chronic radicular pain for at least 3 months and had failed to respond to anti-inflammatory medications, analgesics or physical therapy of at least 4 weeks. Patients with sacroiliac joint or facet joint pain based on clinical or radiological evaluation, psychiatric disorders, bleeding disorders, infection sign, inflammatory disease, or rheumatoid disorders were excluded in this study. Patients who have previous lumbar surgery, progressive motor deficit or significant sensory deficit, cauda equina syndrome were also excluded. We permitted only acetaminophen and nonsteroid anti-inflammatory drugs (NSAIDs) for the pain control.

### Injection Methods

FL- or US-guided CESI to treat unilateral lower lumbar radicular pain due to spinal canal stenosis or herniated disc was a common practice in our service. Patients were informed of the potential risks associated with the procedure, and the benefits and risks of using corticosteroid mixed with contrast media, and then were asked to provide consent. The choice whether to use US or FL was made by the patient.

**FIGURE 1:**
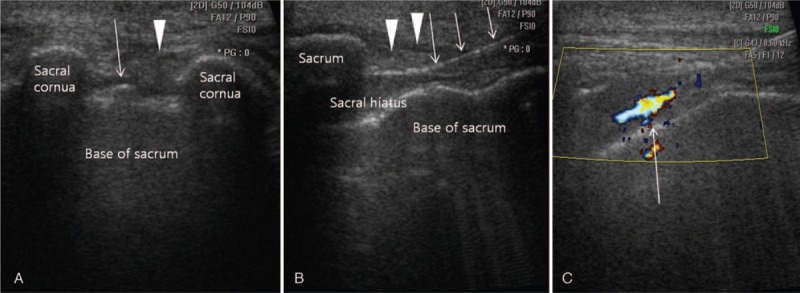
Ultrasound-guided caudal epidural steroid injection. (A) Ultrasound-guided caudal epidural steroid injection in the short axis view. Ultrasound shows the 2 sacral cornus as 2 hyperechoic reversed U-shape structures. The arrowhead is pointing to the sacrococcygeal ligament covering the sacral hiatus. The structure at the bottom is the dorsal bony surface of the sacrum. The arrow indicates the caudal epidural needle. (B) Long axis view showing the needle inside the caudal epidural space. The arrowhead is pointing to the sacrococcygeal ligament. The arrow indicates the caudal epidural needle. (C) Color Doppler Ultrasonography. A predominantly 1-color spectrum is in the caudal epidural space after injection of the contrast media (arrow).

The procedures were performed by 1 physician with >7 years of experience with US- and FL-guided procedures. All the injection procedures were performed as an outpatient clinic setting. We used Accuvix XQ^®^ (Medison, Seoul, Korea) with a linear probe at 6 to 12 MHz as the US instrument. First, US probe was placed transversely at the midline to obtain the sacral hiatus transverse image with the patient in prone position.^7–10^ Two sacral cornua were seen as hyper-echogenic structures with an inverted U shape, and then, sacral hiatus could be identified easily. Before CESI, blood vessels were identified by power doppler imaging. With the help of an assistant, an interventionalist, wearing sterilegloves, set up the equipment needed for the injection on a table covered with a sterile drape. The sacrococcygeal area was prepared using an iodine-based povidone solution and an alcohol solution. The interventionalist then used the middle finger of the dominant hand to localize the tip of the coccyx through palpation. Finally, the interventionalist inserted the needle in the direction of the affected side in order to deliver the medication toward the affected side and to increase the chance of the medication reaching the site of the anomaly.^11^ A spinal needle (Spinocan^®^, BRAUN, Melsungen, Germany) of 22-gauge and 3.5-inch was then inserted into the sacral hiatus through the 2 cornua (Fig. 1A). The US transducer was then rotated 90° to get a longitudinal view of sacrum and sacral hiatus after “pop” or “give” feeling of sacrococcygeal ligament penetration, and then the needle was more advanced into the sacral canal underreal-time US guidance (Fig. 1B).^7–10^

We checked the absence of blood in the syringe before furthering the needle advancement, and then performed an aspiration test to check for the presence of blood and cerebrospinal fluid. If either method identified blood or cerebrospinal fluid, the needle was repositioned. After confirming the absence, first 1 to 2 mL of 1% lidocaine test dose was injected. The flow was observed using color Doppler mode of US (Fig. 1C). We defined a positive spectrum as being observed unidirectional flow of the solution with 1 dominant color through the epidural space beneath the sacrococcygeal ligament, without other directional flow of multiple colors.^10^ If other directional flow was detected, we reset the needle under US guidance and confirmed the needle position by the color Doppler spectrum. The following steps were initiated after monitoring of the onset of clinical manifestations such as mid-back and contralateral lower extremity pain, metallic taste, dizziness, tachycardia, lower extremity paresthesia, auditory changes, slurred speech, and motor ataxia for 1 to 2 min.^11^ And 20 cc of the treatment drug, composed of 20 cc (0.5% lidocaine 18.0 mL + dexamethason 10 mg 2 mL), was injected after confirming the absence of abnormal findings.

For FL-guided CESI, the patients were asked to lie on a fluoroscopic table with their abdomen side down. A pillow was placed under the hips to tilt the pelvis and bring the sacralhiatus into greater prominence. The sacrococcygeal area was prepared using an iodine-based povidone solution and an alcohol solution. The interventionist then localized coccyx tip through palpation with sterilized middle finger. We used a 22-gauge, 3.5-inch length spinal needle (Spinocan^®^, BRAUN, Melsungen, Germany) with the image intensifier. We checked the absence of blood in the syringe before the needle advancement. The inhalation test was performed to check cerebrospinal fluid leakage. We injected ∼1 mL of contrast media (Omnipaque 300; GE Healthcare, Carrigtohill, Co. Cork, Ireland) before drug injection to confirm epidural flow and to avoid intravascular, intradural, or soft tissue infiltration.

First, we injected 1 to 2 mL of 1% lidocaine as a test dose and monitored for any clinical symptoms such as mid-back and contralateral lower extremity pain, metallic taste, dizziness, tachycardia, lower extremity paresthesia, auditory changes, slurred speech, and motor ataxia for 1 to 2 min (12). We injected 20 cc of the treatment drug (0.5% lidocaine 18.0 mL + dexamethason 10 mg 2 mL) in the absence of such abnormal findings.

In both groups, patients received 2 consecutive injections 2 weeks apart. The second injection proceeded conditionally. If the initial injection resulted in significant symptom reduction (VNS ≥ 50%), the second injection was omitted with a progression to follow-up. If no pain relief or pain deterioration was observed, a second injection or re-evaluation was not considered. If the patients experienced pain relief of <50% reduction in VNS, a second injection was scheduled. Because none of the patients had shown any improvement by medications such as anti-inflammatory drugs and physical therapy for 4 weeks, we did not set any limit on the continuation of previous exercise programs, drug therapy, and work. There were no specific additional interventions.

### Review of the Clinical Data

**FIGURE 2:**
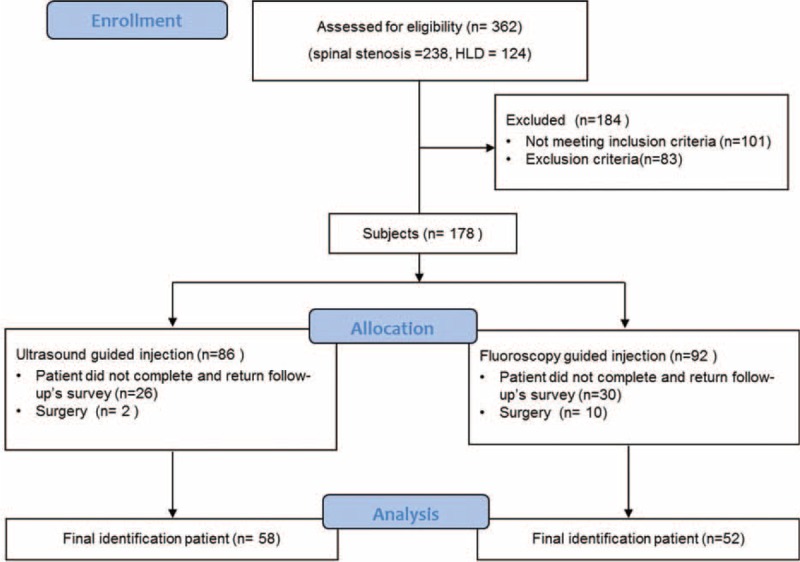
Subjects flow diagram.

Of the 178 CESIs, US (n = 86) or FL (n = 92) performed during the interval encompassed by this study, the inclusion criteria were met for 110 (62%) injections. Fifty-six (31%) injections were excluded because the patient did not complete and return follow-up's survey. Twelve (7%) of the injections were excluded because of exclusion criteria. Finally, 58 patients in the US group and 52 patients in the FL group were left in this study (Fig. 2). A standardized chart abstraction form was used to extract collected demographic data, treatments, pain severity, and function assessment. Follow-up interviews were performed by a nursing personnel not involved in the procedure. The VNS and ODI were used to evaluate the clinical effectiveness in terms of pain reduction and functional improvement at pretreatment, 3, 6, and 12 months after the last injection. On the VNS, a score of 0 indicates no pain, and a score of 10 indicates the worst pain imaginable, in whole numbers with 11 integers including zero.^13^ ODI is one of the most commonly used disease-specific measures for patients with LBP.^14^ ODI ranges from 0 to 100 is one of the most common measures when we assess outcome of LBP patients.^14^ ODI is composed of 10 items. Each item is scored from 0 to 5, and the total score is calculated by added of each item and multiplied by 2.

Independent variables such as injection method, number of injections, cause of radicular pain (spinal stenosis or herniated lumbar disc), pain duration, sex, and age were from medical records. Predictive variables were measured as follows:

We grouped patients as 5 according to their age: <39 years old, 40 to 49, 50 to 59, 60 to 69, and >70. The duration of radicular pain was analyzed as a potential predictive value, and classified as acute of subacute (< 6 months) or chronic (≥6 months).^15^

We defined effective treatment as a reduction in the VNS score of 50% or more, and ODI of 40% or more at 3, 6, and 12 months.^16^ Patients who did not satisfy these conditions were considered as ineffective treatment. Patients who were treated by repeated CESI or surgical treatment were also considered as ineffective treatment.

We checked immediate adverse events such as vasovagal reaction, facial flushing, or severe back pain within a few minutes after the injection. Every patients were asked to complete a questionnaire within 48 h. After the procedure to evaluate the occurrence of side effects including headaches, fever, transient pain exacerbation, hematoma, or loss of control of heart disease, hypertension, or diabetes.

## STATISTICS

The sample size was calculated based on significant pain relief. Considering a 2-sided significance level of 0.05, a power of 80%, and an allocation ratio of 1:1, it was estimated that 55 patients were required for each group.^17^ Previous studies of interventional techniques identified 50 to 60 patients as appropriate.^18–20^

Pearson's chi square test was used to compare the characteristics of the 2 groups in variables such as sex difference, target nerve root, cause, and number of injection. Mann–Whitney *U* test was used to evaluate for age, body mass index (BMI), and pain duration difference between the 2 groups. Repeated measure analysis of variance (ANOVA) was done to compare VNS and ODI at each time point and Bonferroni's correction for post-hoc comparison. The chi square test was used to analyze the correlation of possible outcome predictors with the therapeutic effect. Logistic regression modeling was performed to determine the influence of injection methods, patients’ age, gender, and pain duration on successful outcome. Statistical analyses were performed using SAS Enterprise Guide 4.1. The level of statistical significance was set as *P* < 0.05.

## RESULTS

The mean age of the patients was 56.3 ± 9.7 in the FL group and 57.9 ± 9.9 in the US group, without a significant difference. No significant differences were observed in the general characteristics of sex, BMI, pain duration, injection number, lesion location, and topographical comparisons (Table 1).

**TABLE 1:**
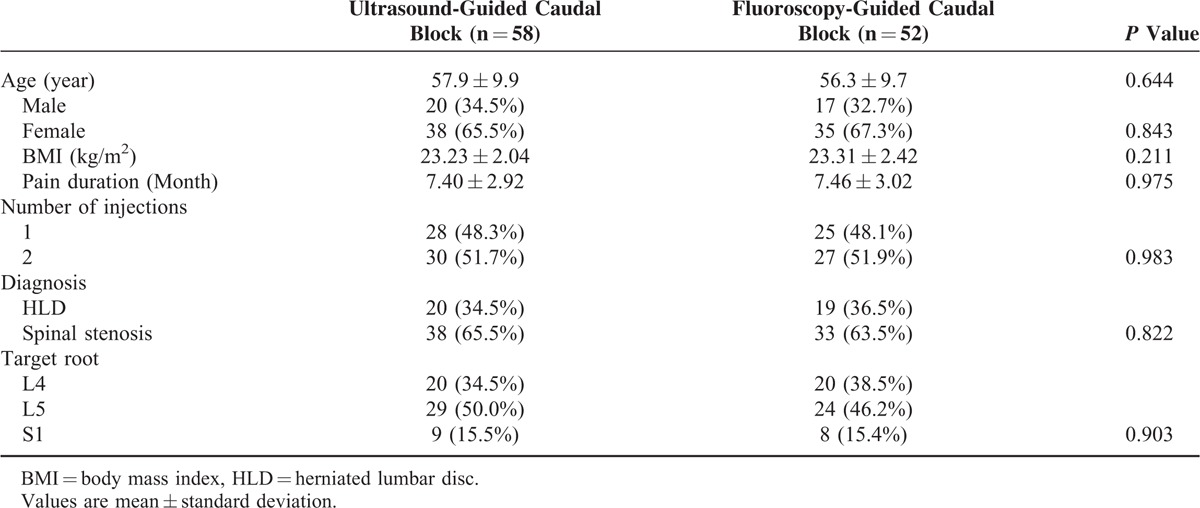
General Characteristics of the Patients

ODI and VNS showed significant improvement at 3, 6, and 12 months after the last injections in both groups. No significant differences in ODI and VNS at the point of baseline, 3, 6, and 12 months after the last injections were observed between the groups (Table 2).

**TABLE 2:**
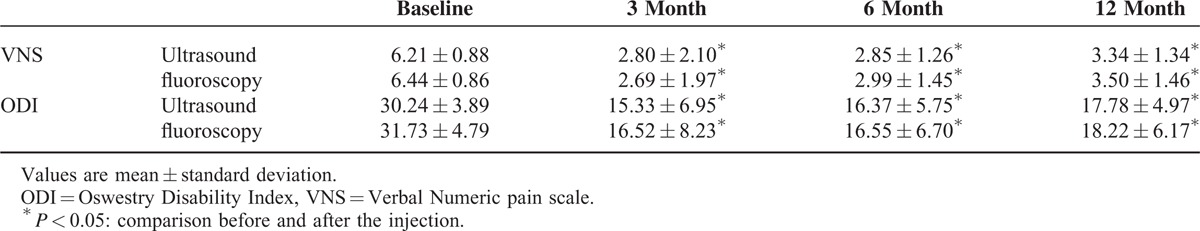
Comparison of VNS and ODI from Baseline to 3, 6, and 12 Months After Last Injection

**FIGURE 3:**
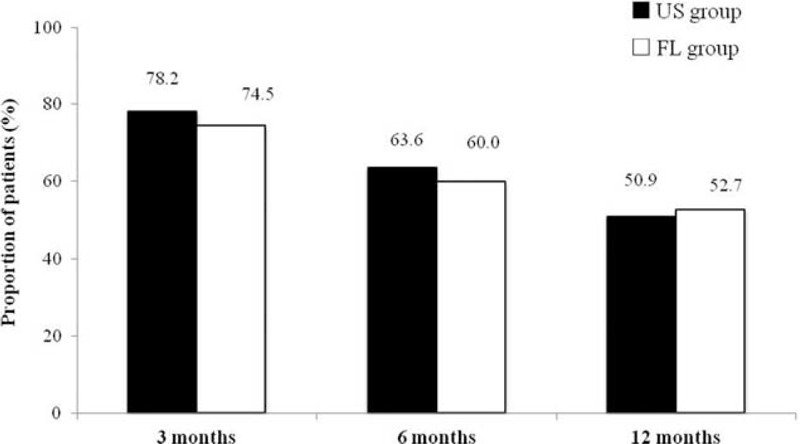
Illustration of significant pain relief (≥ 50% reduction in the verbal numeric pain scale from baseline) and functional improvement (≥ 40% reduction in the Oswestry Disability Index from baseline). FL group = fluoroscopy-guided caudal block, US group = Ultrasound-guided caudal block.

The proportion of patients with >50% improvement in the VNS score and >40% improvement in the ODI is illustrated in Figure 3, showing 53.4% in US groups and 51.9% in FL groups at 12 months. There were no significant differences between the groups at the 3-month to 6-month to 12-month periods.

Injection method, gender, age, pain duration, cause, and number of injection were not found to be statistically related to the effectiveness of CESI. Injection method, gender, age, pain duration, cause, and number of injection were not independent predictors of effectiveness of CESI by using univariate and multiple logistic regression analyses(*P*>0.05). These results are summarized in Tables 3 and 4.

**TABLE 3:**
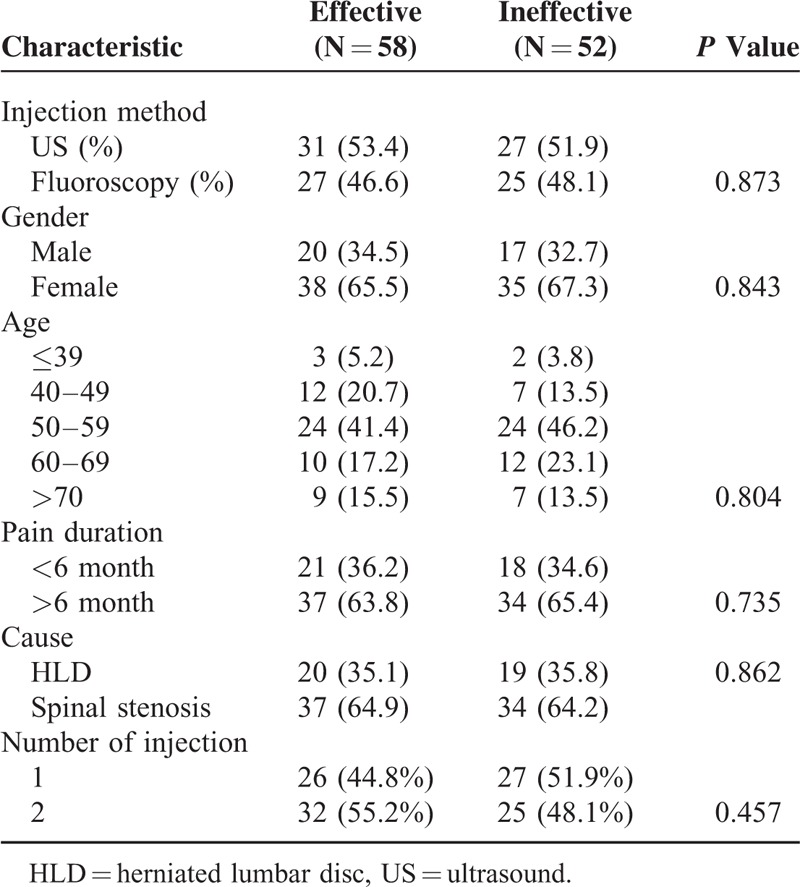
Univariable Analysis for Possible Outcome Predictors for Injection Effectiveness at Follow-Up

**TABLE 4:**
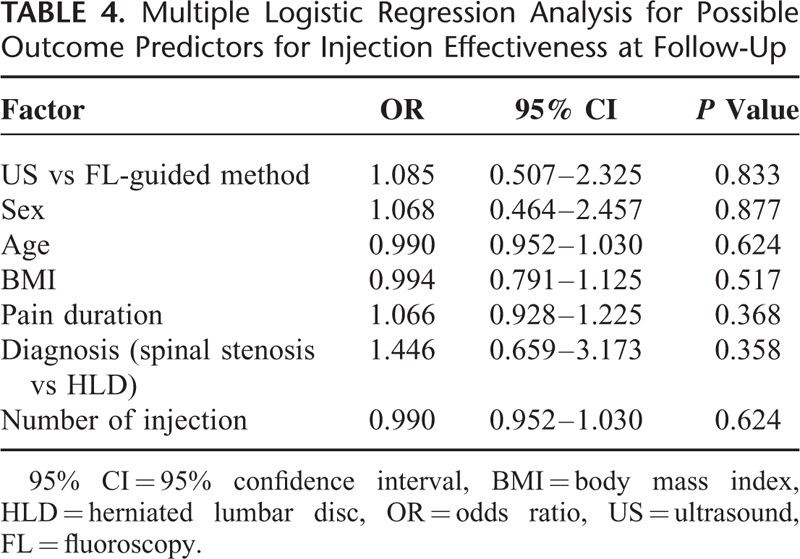
Multiple Logistic Regression Analysis for Possible Outcome Predictors for Injection Effectiveness at Follow-Up

Two patients in the US group and 3 in the FL group reported a vasovagal reaction immediately after the procedure with no statistical difference between groups. Three patients in the US group and 2 in the FL group reported a transient headache with no statistical difference. Overall, among injected patients, 5 patients in the US group and 4 in the FL group reported transient back or lower limbs pain exacerbation within 48 h after the procedure. No patient reported headache suggestive of post lumbar puncture syndrome, decompensated heart disease, and diabetes. No instances of infection or hematoma were recorded during the 2 weeks period after the procedure.

## DISCUSSION

This retrospective study assessing treatment effects of CESI with US or FL guidance showed clinically meaningful and significant improvement in all parameters at the end of a 1-year period. We demonstrated successful treatments (≥50% improvement in the VNS score and ≥40% improvement in the ODI) in 53.4% to 51.9% of patients in both groups at the end of the 1-year period. Successful treatments did not differ significantly between the groups or from the 3-month to 6-month to 12-month outcomes. Consequently, pain reduction and functional improvement were similar for US-guided CESI and FL-guided CESI.

Various experimental studies have shown that radicular pain occurs as a result of both mechanical compression and chemical radiculitis.^21–23^ Therefore, local delivery of corticosteroid with both anti-inflammatory and local anesthetic properties to the affected nerve root appears to be a reasonable option.^23–25^ The epidural corticosteroid injection may be classified as interlaminar, caudal, or transforaminal, depending on the approach to the epidural space.^9^ CESI is an easy and safe way to administer drug in the outpatient setting with a lower risk of thecal sac puncture and intrathecal injection.^7,26^ However, inaccurate needle location has been reported in 25% to 36% of cases in unaided procedures.^4,27^ The FL-guided procedure is commonly used for the spinal intervention procedures and recommended for the confirmation of the correct caudal epidural needle placement.^28,29^ But the radiation exposure has been the major concern in clinical fields. The advantages of US are that it is easy to use, radiation-free, and can be used in virtually any clinical setting.^30^ Most significantly, US can provide real-time and continuous needle guiding images without radiation exposure.^3^

Klocke et al ^7^ first reported the use of US during CESI and particular advantages in moderately obese patients or patients with difficulty in prone positioning. Chen et al,^3^ who evaluated US-guided CESI in 70 patients with radicular pain, reported a 100% success rate in needle placement using a high-frequency transducer to identify the sacral hiatus during injection. In addition to accuracy and feasibility, US can provide clear images of the sacral hiatus and detect the anatomic variations of the sacrum and sacral hiatus that make CESI difficult or impossible.^26^ In the previous studies, ∼2% to 3% of the studied population have closed sacral canals, thus making CESI impossible for these subjects.^27,31^ Measurement of the diameter of the sacral canal at the apex of the sacral hiatus is also important. Sekiguchi et al ^31^ reported that the diameter of the sacral canal was <2 mm in 1% of cases. Another study reported such a diameter in 6.25% of cases.^32^ In cases of <2 mm, patients complained of severe soreness and pain at the injection site when CESI was attempted after the injection needle just penetrated through the sacrococcygeal ligament.^27^ Strong resistance was felt during the injection procedure, and injection of the medication into the sacral canal was nearly impossible.^27^ Additionally, a needle >22 gauge may cause insertion failure through the sacreal hiatus in the case of narrow sacral hiatus <2 mm diameter. Chen et al ^27^ reported 4 cases of injection failure when the sacral hiatus diameter was between 1.2 and 1.6 mm. Park et al ^9^ reported 4 cases of injection failure with FL-guided CESI and all of their sacral hiatus diameters measured by US were under 2 mm.^9^ Thus, obtaining of anatomical information such as sacra hiatus shape and diameter before CESI is important in terms of reducing patient discomfort and saving procedure time.

Despite widespread use and numerous publications there is controversy with regards to the medical necessity and indications for lumbar epidural injections. Multiple previous studies have identified indications for caudal epidural injections in positive reports to treat radicular pain from herniated lumbar intervertebral disc or spinal stenosis.^33–35^ Manchikanti et al ^33^ reported that CESI led significant pain relief (≥50%) in 55% to 65% of patients with LBP due to spinal stenosis after CESI. Botwin et al^34^ reported 65% of patients showed a successful outcome (≥50% reduction in visual analog pain score) in 6 weeks after CESI, 62% in months, and 54% in 12 months. In cases of radicular pain due to disc herniation, Manchikanti et al^35^ reported proportion of patients with significant pain relief of 50% or greater and/or improvement in functional status with 50% or more reduction in ODI scores of 77% and 75% in patients receiving steroids with average number of procedures per year of 3.8. As illustrated in the present study, the proportion of patients with successful outcome is showing 53.4 % in US groups and 51.9 % in FL groups at 12 months.

Compared to the FL-guided procedure, the US-guided procedure has several limitations. Faulty injections resulting in low effectiveness cannot be detected during the US-guided procedures. In the case of unilateral radicular pain, a diminished therapeutic effect could be expected, if a drug might be administered to the opposite site. On the other hand, FL-guided procedure has advantage that adjusting needle direction is possible in the case of detecting incorrect drug administration. In order to overcome the limitation of the US-guided procedure, experts in CESI have recommended inserting the needle in the direction of the affected side in order to deliver the medication toward the affected side and to increase the chance that the medication will reach the site of the anomaly.^11,35^ Despite using this method, Lee et al ^36^ reported that 13.6% of the studied patients had a greater amount of the drug on the opposite side of the lesion. Positioning the patient in the lateral decubitus on the side of their leg pain results in accumulation of the injected drugs on the dependent side due to gravity and can solve the problem. Makki et al ^37^ reported that laying a patient on the side of their leg pain after CESI has a beneficial effect on the degree of pain relief. We expect that the combination method of needle insertion direction to the affected side and patient positioning can result in a more accurate US-guided procedure.

A second limitation is that the inadvertent intravascular injection cannot be detected during US-guided CESI. The frequency of accidental intravascular injections during CESI was reported to range from 2.5% to 9%.^4,38^ The needle aspiration test before drug injection is helpful for the prevention of intravascular injection, but is neither sufficiently sensitive nor specific to avoid an intravascular needle position.^9^ Color Doppler and lidocaine test dose injections were used to protect intravascular injections. According to Yoon et al ^10^ if the injection flow was not detected as being mainly in the cephalic direction and vascular flow was detected by the Doppler as having multicolored spectrums, it was considered that the medication was injected into the vessel. In such cases, the needle tip must be relocated. Using such an identification method, Yoon et al ^10^ reported a successful injection rate of 94% and in 3 other failure cases, they presumed that the cause was positional changes of the needle after exchanging the syringe.^10^ Park et al ^9^ used live FL during injection of 1 to 2 cc contrast media with color Doppler mode. They could obtain an accurate contrast media dye position in the epidural space and no case of intravascular injection in all 60 subjects by using the color Doppler spectrum confirmation method during live FL.^9^ It reveals that the application of color Doppler during the injection procedure could significantly reduce the intravascular injection rate. In addition, we recommend lidocaine test dose injections to rule out the possibility of intravascular injections. Using 1cc, 1% lidocaine as an anesthetic test dose was recommended for the prevention of intravascular drug injection during the transformianal injection.^12^ We also used 1 cc, 1% lidocaine as a test dose before drug injections. Patients were monitored for any special reaction for 1 to 2 min after the test dose injections before proceeding to the actual treatment injections. Although positive findings of intravascular injection were not observed, a confirmation step with the test dose provided reassurance regarding the absence of intravascular injections.

The present study had several limitations. First, this study was retrospective in design. Although subjects were selected according to the inclusion and exclusion criteria, there could have been heterogeneity among the subjects included in this study. In addition, other treatments such as medication or physical therapy during follow-up period might have influenced to outcomes. However, we thought that these effects were very limited because patients refractory to these treatments were included in this study. Second, both procedures were performed by 1 physician, reflecting the experience of 1 practitioner and limiting the generalization of the study results. Third, the US-guided approach was performed in patients with a BMI <30 kg/m^2^ in this study. Since visualization of small vessels or radicular arteries by US may be difficult in obese patients, there can be some different results in the study including obese patients. Additional studies may be required in order to improve such limitations of this study.

In conclusion, pain reduction and functional improvement were similar for the US-guided procedure and the FL-guided procedure without the risk of radiation exposure. Therefore, by considering our data from this retrospective study, US-guided CESI is deserving of consideration for conservative management of unilateral lower lumbar radicular pain.

## CONCLUSION

In this study, US-guided CESI showed similar improvement in pain relief and function as the FL-guided injection for the treatment of unilateral lower lumbar radicular pain. Therefore, based on our data from this retrospective study, US-guided CESI deserves to be considered among the conservative managements of unilateral lower lumbar radicular pain.
